# Non-coding variants disrupting a tissue-specific regulatory element in *HK1* cause congenital hyperinsulinism

**DOI:** 10.1038/s41588-022-01204-x

**Published:** 2022-11-04

**Authors:** Matthew N. Wakeling, Nick D. L. Owens, Jessica R. Hopkinson, Matthew B. Johnson, Jayne A.L. Houghton, Antonia Dastamani, Christine S. Flaxman, Rebecca C. Wyatt, Thomas I. Hewat, Jasmin J. Hopkins, Thomas W. Laver, Rachel van Heugten, Michael N. Weedon, Elisa De Franco, Kashyap A. Patel, Sian Ellard, Noel G. Morgan, Edmund Cheesman, Indraneel Banerjee, Andrew T. Hattersley, Mark J. Dunne, International Congenital Hyperinsulinism Consortium, Sarah J. Richardson, Sarah E. Flanagan

**Affiliations:** 1 Institute of Biomedical and Clinical Science, University of Exeter Medical School, UK; 2 Exeter Genomics Laboratory, Royal Devon and Exeter NHS Foundation Trust, Exeter, UK; 3 Endocrinology Department, Great Ormond Street Hospital for Children, London, UK; 4 Department of Paediatric Pathology, Royal Manchester Children’s Hospital, Oxford Road, Manchester, UK; 5 Department of Paediatric Endocrinology, Royal Manchester Children’s Hospital, Oxford Road, Manchester, UK; 6 Faculty of Biology, Medicine and Health, The University of Manchester, Oxford Road, Manchester, UK

## Abstract

Gene expression is tightly regulated with many genes exhibiting cell-specific silencing when their protein product would disrupt normal cellular function^1^. This silencing is largely controlled by non-coding elements and their disruption might cause human disease^2^. We performed gene-agnostic screening of the non-coding regions to discover new molecular causes of congenital hyperinsulinism. This identified 14 non-coding *de novo* variants affecting a 42bp conserved region encompassed by a regulatory element in intron 2 of *Hexokinase 1* (*HK1*). HK1 is widely expressed across all tissues except for liver and pancreatic beta-cells and is thus termed a “disallowed gene” in these specific tissues. We demonstrated that the variants result in a loss of repression of *HK1* in pancreatic beta-cells, thereby causing insulin secretion and congenital hyperinsulinism. Using epigenomic data accessed from public repositories, we demonstrated that these variants reside within a regulatory region that we determine to be critical for cell-specific silencing. Importantly, this has revealed a disease mechanism for non-coding variants that cause inappropriate expression of a disallowed gene.

Genetic discovery in Mendelian disease has focused on identifying highly penetrant variants affecting the function of genes expressed in clinically affected tissue(s). Whilst this approach has proven successful the underlying aetiology of over 3000 presumed monogenic diseases remains undefined^2–5^, of which many have significant clinical and genetic heterogeneity^6,7^. Congenital Hyperinsulinism (CHI), is characterised by inappropriate insulin secretion during hypoglycaemia. It is a clinically and genetically heterogeneous disease where, despite extensive sequencing efforts, the underlying aetiology is not known in up to 50% of individuals^8,9^.

To identify new aetiologies, we performed whole-genome sequencing (WGS) on 135 individuals with biochemically confirmed, persistent CHI without an identified disease-causing variant in a known gene (Supplementary Table 1). We initially searched for genes containing new coding *de novo* or biallelic variants in two or more individuals. When no such genes were found, we turned to the non-coding genome and searched for *de novo* copy number variants. This identified a single locus within intron 2 of Hexokinase 1 (*HK1*) containing two heterozygous ~4.5Kb deletions with a 2,787bp overlap in two unrelated individuals (Extended Data Figure 1). Digital droplet PCR confirmed the deletions in both probands and an affected twin.

We next searched for *de novo* single nucleotide variants and indels within the minimal deleted region within our whole genome sequencing discovery cohort and in a replication cohort of 27 individuals with CHI who had undergone pancreatectomy as part of routine clinical care (Supplementary Table 1). This identified 7 different *de novo* variants in 12 probands. In two cases the variants had been inherited by similarly affected offspring (Figure 1). All variants were new^10,11^ and within a 42bp conserved region constrained against variation in gnomAD.^12^

Overall, the finding of 9 different *de novo* variants in 14 probands, co-segregating with disease in three additional family members provides overwhelming evidence of disease-causality.

All 17 individuals with a *HK1* non-coding variant had severe early-onset CHI (median age at diagnosis: Birth [IQR: 0-14 d]). These variants were not associated with macrosomia at birth (median birthweight z score: 0.61 [IQR: 0.10-1.84]). This suggests that insulin secretion was not markedly increased *in* utero given its role as a potent growth factor during fetal development. In all cases the hyperinsulinism persisted, the eldest individual still known to be affected was 18 years of age. These findings suggest that the variants act to disrupt glucose-induced insulin secretion throughout post-natal life and into adulthood but have less impact during fetal development.

The *HK1* variants caused a beta-cell specific defect with no common additional features between patients. In five individuals, medical management was ineffective, leading to pancreatic resection. Of these, the only case that had resolution of the CHI developed insulin-dependent diabetes following near-total pancreatectomy. Histopathological analysis of resected pancreatic tissue (n=2) demonstrated a discrete pathology, distinguishable from diffuse disease resulting from *ABCC8* variants, the commonest cause of CHI (Extended Data Figure 2). An overview of the clinical features is provided in Supplementary Tables 2-3.

*HK1* encodes a glycolytic enzyme that is silenced in the pancreas and liver but expressed in all other mature tissues (Extended Data Figure 3a-c) where it supports glucose metabolism, ensuring cell survival^13^. Within the pancreatic beta-cell, glucose is phosphorylated to glucose-6-phosphate by Glucokinase (GCK; Hexokinase 4) which, due to a low-binding affinity (Km ~8mM) acts as the pancreatic glucose-sensor coupling insulin release to the prevailing glucose concentration^14^. Hexokinase 1 has a markedly higher affinity for glucose (Km <50µM)^14^. Silencing of *HK1* in favour of *GCK* in the beta-cell therefore ensures appropriate glucose-sensing, minimising insulin release at low glucose levels. Whilst bi-allelic loss-of-function *HK1* variants have been reported to cause non-spherocytic haemolytic anaemia^15^, dominant or recessive coding variants have not been described in individuals with defects in glucose homeostasis; in keeping with the absence of HK1 protein in beta-cells.

In resected pancreatic tissue of individuals with non-coding variants, we found that HK1 was expressed and co-localised with insulin in the islets of affected tissue but not in controls (Figure 2). HK1 did not co-localise with glucagon (secreted by pancreatic alpha-cells) suggesting that the impact of the variant was beta-cell specific (Extended Data Figure 4). These results confirmed that the variants cause HK1 to be inappropriately expressed in the pancreatic beta-cell and explain why there is increased insulin secretion in these individuals during hypoglycaemia.

We identified a putative islet cis-regulatory domain encompassing our critical region^16^ bound by a broad set of islet transcription factors, with NKX2-2 and FOXA2 most prominent (Figure 3). Single-nuclei ATAC-seq (snATAC-seq) data in human islets^17^ revealed a peak of open chromatin in beta-cells and showed that the relevant *HK1* promoter remains accessible (Figure 3b). Human islet Hi-C data^18^ confirmed that both our region and the relevant *HK1* promoter are contained within a well-insulated domain (Extended Data Figure 5), without evidence of contact to distal loci (Figure 3d, Extended Data Figure 5b) further supporting direct regulation by the critical region on the promoter.

During development, HK1 is highly expressed in embryonic stem cells but is progressively downregulated during pancreatic cell-type differentiation becoming extinguished during beta-cell maturation^19,20^ (Figure 3a, Extended Data Figure 3d). GCK shows increased expression during differentiation as HK1 is downregulated (Figure 3a). Despite being absent from beta-cells, HK1 is expressed in mature stellate- and duct-cells suggesting differential regulation across pancreatic cell-types (Extended Data Figure 3e).

Chromatin accessibility data shows our critical region remains closed over pancreatic-cell differentiation until pancreatic progenitor stages, when it becomes accessible and bound by a complement of factors in the pancreatic progenitor regulatory network^21,22^ (Figure 3c, Extended Data Figure 6b-c). This suggests the region is necessary for HK1 repression in late pancreatic development and mediating beta-cell specific control in adult cells but that it does not have a role in the reduction of HK1 expression between embryonic stem cells and early pancreatic progenitors (Figure 3a).

Analysis of histone modifications during pancreatic differentiation, in human islets^21,23^, cell sorted human pancreatic alpha, beta and exocrine cells^24^ and the EndoC-βH1 beta-cell line^25^, revealed that repression is actively maintained. The critical region is marked by a bivalent state encompassed by focal peaks of active enhancer marks H3K4me1 and H3K27ac and a broader and dominant polycomb H3K27me3 repressive domain (Extended Data Figure 6d). This domain is diminished in exocrine cells supporting that this mode of repression is specific to endocrine cell types (Extended Data Figure 5d).

Analysis of transcription factor motif families revealed that the variants disrupt transcription factor binding sites for FOX, NKX2 and NFAT families (Supplementary Table 5, Figure 3e), which each have family members expressed in beta-cells (Extended Data Figure 7). This is supported by ChIP-seq data revealing that NKX2-2 and FOXA2 were prominently bound at this region in islets (Figure 3b). FOXA2 loss-of-function causes CHI^26^, and NKX2-2 is able to recruit a large repressive complex that regulates beta-cell specification through DNA methylation^27^ and may therefore have a role in maintaining the repressive epigenetic state at the *HK1* locus^21,23–25,28^. Multiple members of the NFAT family are expressed in beta-cells (Extended Data Figure 7), with NFATC2 inducing beta-cell proliferation in human islets^29^. Analysis of snATAC-data from human islets revealed endocrine cells present in hormone-high and hormone-low states^17^, with the former associated with increased promoter accessibility over secreted endocrine genes and the latter over cell-cycle genes. Our critical region is accessible in the hormone-high state of alpha, beta, and delta-cells, and when compared between hormone-low state cells, only remains accessible in beta cells (Extended Data Figure 6a). This suggests that HK1 repression is actively maintained during beta-cell proliferation and not during alpha or delta-cell proliferation, which given NFATC2’s capacity to induce beta-cell proliferation, supports this factor in maintaining HK1 repression.

Discovering non-coding regulatory variants controlling *HK1* and access to a broad range of publicly available epigenomic data^16–25,30,31^ enabled us to identify a regulatory element critical for the selective silencing of an otherwise ubiquitously expressed gene within a single cell-type. It is interesting to note that interruption of transcription factor binding is implicated in each of the 9 distinct variants and the locus is decorated by both active and repressive epigenetic marks suggesting that continual transcription factor binding is necessary for maintenance of repression in beta-cells. Future work should prioritise regulatory regions surrounding disallowed genes with similar epigenetic marks and to explore whether different regulatory mechanisms exist to silence disallowed genes within the beta-cell.

Over 60 beta-cell disallowed genes have been described, although the mechanism(s) controlling cell-specific silencing in humans have not been fully determined^1,32^. Linkage analysis of *HK1* to CHI^33^ and HK1 expression in beta-cells^34^ have been reported in several patients, but the genetic aetiology was not established. Promoter variants in the beta-cell disallowed gene, *SLC16A1* have also been reported in two families with exercise–induced hyperinsulinism. However, these variants increased transcription across cell types leading to the hypothesis that differences in the post-transcriptional regulation of mRNA across tissues could explain the beta-cell-specific phenotype^35^.

The identification of non-coding *HK1* variants in individuals with CHI represents a rare example of regulatory non-coding variants affecting a gene in which coding variation does not cause the same phenotype^36^. These findings highlight a role for undiscovered regulatory variants causing disease through inappropriate expression of a normally functioning protein in a specific cell-type.

Non-coding variants affecting the regulation of *HK1* cause CHI through its aberrant expression in beta-cells. These findings are important for future efforts to discover non-coding regulatory variants as they establish a critical role for disallowed genes in Mendelian disease.

## Methods

### Subjects

Congenital hyperinsulinism (CHI) was defined as an inappropriately high level of plasma insulin at the time of hypoglycaemia associated with inappropriately supressed ketones and free fatty acids presenting within the first 12 months of life. The definition of hypoglycaemia was based on the recommendations of the Pediatric Endocrine Society (blood glucose <2.8mmol/L in the presence of detectable insulin)^37^. Clinical details of the subjects are provided in Supplementary Table 1. Subjects with CHI were recruited by their clinicians for molecular genetic analysis to The Exeter Genomics Laboratory. Disease-causing variants in the known CHI genes had been excluded by targeted-next generation sequencing in all cases^38^.

### Ethical considerations

This study was approved by the North Wales Research Ethics Committee (17/WA/0327, IRAS project ID: 231760) and was conducted in accordance with the Declaration of Helsinki, with all subjects or their parents providing informed consent for genetic testing. All tissue samples were studied with full ethics approval (West of Scotland Research Ethics Committee, reference: 20/WS/0074 IRAS project ID: 283620 or nPOD)^39^. No participant received compensation for entering this study.

### Sample collection and DNA Extraction

Peripheral whole blood samples (1-5mls) were taken in EDTA blood collection tubes. Automated DNA extracted was undertaken on a Chemagic STAR (Hamilton Bonaduz AG, Switzerland) using the Chemagic STAR DNA blood extraction kit (CMG-1756, [Perkin Elmer, Waltham,USA]).

### Whole-genome sequencing

Whole-genome sequencing of DNA extracted peripheral blood leukocytes was completed on 135 probands with CHI (n=3 with Illumina HiSeq 2500, n=69 with Illumina HiSeq X10, n=63 with BGISeq-500). The mean read depth across the whole genome was 36.9 (stddev 4.9). An additional 191 family members were also sequenced. The sequence data was aligned using BWA MEM version 0.7.15, and processed using a pipeline based on the GATK best practices (Picard version 2.7.1, GATK version 3.7). Variants were annotated using Alamut batch standalone version 1.11 (Rouen, France).

CNVs were called across the genome by SavvyCNV^40^ using a bin size of 2Kbp. We also developed a new tool (FindLargeInsertSizes, available at https://github.com/rdemolgen/SavvySuite) to detect small (≥1Kbp) deletions, insertions, inversions, and translocations using read pair information. We used this tool to screen all whole genome sequenced samples for structural variants within *HK1* intron 2.

When a variant was identified, Sanger sequencing (for single nucleotide variants/indels) or digital droplet PCR (for deletions) was performed on samples from each available family member to confirm genome sequencing results.

**Digital droplet PCR (ddPCR):** was performed on leukocyte DNA from patients 1 and 2.1 and their unaffected parents to confirm the results of the whole-genome sequencing deletion analysis. ddPCR was also performed on the affected twin brother of patient 2.1 and the 27 individuals with CHI of unknown cause who had undergone pancreatectomy.

Relevant primers (Supplementary Table 4) were used in reactions for droplet generation, PCR and detection using the Bio-Rad QX200 ddPCR EvaGreen system (Hercules, CA, USA) to search for copy number changes. Reactions were performed as per the manufacturer’s recommendations with an annealing/extension temperature during PCR of 59°C. All data were analysed using Bio-Rad QuantaSoft software version v1.6.6.0320.

### Sanger sequencing of the *HK1* regulatory element

Sanger sequencing was performed on DNA extracted from peripheral blood leukocytes from 27 individuals with CHI of unknown cause who had undergone pancreatectomy. Briefly, a 397bp genomic region (GRCh37/hg19, Chr10:71,108,536-71,108,932) within intron 2 of *HK1* was amplified by PCR (primer sequences are listed in Supplementary Table 4). PCR products were sequenced on an ABI3730 capillary machine (Applied Biosystems, Warrington, UK) and analysed using Mutation Surveyor v3.24 software (SoftGenetics, State College, PA, USA). When a variant was identified, samples from family members were tested to investigate co-segregation and microsatellite analysis using the PowerPlex kit (Promega, Southampton, UK) was performed to confirm family relationships.

### Histopathology

Formalin-fixed paraffin-embedded pancreatic tissue was available for immediate analysis from two individuals with *HK1* variants (patients 1 and 6, Supplementary Table 2) age-matched non-HI controls (n=2) and age-matched tissues from individuals with HI due to an *ABCC8* variant (HI controls n=2). Immunohistochemistry was performed on 5-µm-thick sections of tissue^41^. For high-content quantification of tissue, sections were first digitized using the 3DHistech Pannoramic 250 Flash II slide scanner (3DHISTECH, Budapest, Hungary) and quantified using QuPath (available at https://qupath.github.io)^42^.

### Immunofluorescence

After dewaxing and rehydration, pancreatic samples from HK1 patients 1 and 6 (Supplementary Table 2) and 4 age-matched control tissues (HI controls (individuals with *ABCC8* variants (n=2); non-hyperinsulinism donors (n=2)) were subjected to heat–induced epitope retrieval (HIER) in 10mM citrate pH6 buffer for 20 minutes. After blocking, the sections were probed in a sequential manner with rabbit monoclonal anti-hexokinase 1 (Abcam ab150423; 1/100 overnight), followed by detection with goat-anti-rabbit Alexa Fluor™ 488 (Invitrogen, Paisley, U.K). Following washes, sections were then probed with mouse monoclonal anti-glucagon (Abcam ab10988; 1/2000 for 1h) followed by detection with goat-anti-mouse Alexa Fluor™ 555. Finally, sections were probed with guinea-pig anti-insulin (Agilent; C#IR002; 1/5 for 1h), followed by detection with goat-anti-guinea-pig Alexa Fluor™ 647 in the presence of DAPI (1µg/ml) to identify cell nuclei. Sections were not stripped between successive antibody incubations. After mounting, the sections were imaged via a Leica DMi8 confocal microscope (Leica Microsystems UK, Milton Keynes, UK) (using a focal plane of 0.4µm) and the distribution of HK1, insulin and glucagon examined in multiple islets. In addition, sections were scanned at 40x magnification using an Akoya Biosciences Vectra^®^ Polaris™ Automated Quantitative Pathology Imaging System for quantification analyses. The quantification of the HK1 expression in islets was determined using the Random Forest Classifier Module (Version 3.2.1851.354), DenseNet AI V2 and HighPlex FL v4.04 modules included in Indica Labs HALO Image analysis platform (Version 3.2.1851.354). The Random Forest Classifier was used to identify the islets, then the DenseNet AI V2 module was used to identify beta and alpha cells within the islets. This enabled exclusion of HK1 positive non-islet cells such as red blood cells, stellate cells and endothelial cells which are frequently found in proximity to the islet. The median fluorescence intensity (MFI) of HK1 expression in beta and alpha cells were then calculated using the HighPlex FL v4.04 module. This pipeline was applied to all corresponding tissue sections. GraphPad Prism V9.2.0 (332) was used to demonstrate the median and IQR of beta and alpha cells MFI in each of the donors assessed.

### Epigenomic analysis

All published ChIP-seq, ATAC-seq, RNA-seq and scRNA-seq datasets used were obtained from accessions provided in Supplementary Table 5. All code and source data required for all analysis, including to determine disrupted motifs and generate figures is provided in https://github.com/owensnick/HK1FigureNotebook.jl and DOI:10.5281/zenodo.6815326.

### Public Bulk ChIP and ATAC-seq

ChIP-seq and ATAC-seq reads aligned with Bowtie2^43^ to the GRCh37/hg19 genome with Bowtie2 v2.3.5.1 with default parameters for single end reads and with additional options “-I 0 -X 1000 --no-discordant --no-mixed” for paired-end reads. Alignments were filtered for those with mapping quality > 30 and then reads with identical aligning coordinates were treated as duplicates and collapsed to a single alignment. For islet H3K27me3 data from roadmap epigenomics project, aligned reads were downloaded in BED/TagAlign format^23^. All data is visualised as reads per million using https://github.com/owensnick/GenomeFragments.jl. For human islet single-nuclei ATAC-seq data^17^ (GSE160472), reads from the authors’ combinatorial barcode approach were aligned as above and separated into cell types using author provided cluster labels: https://github.com/kjgaulton/pipelines/tree/master/islet_snATAC_pipeline.

Human islet Hi-C data was obtained from experiment accession TSTSR043623 and file accession DFF064KIG (.hic file) and TSTFF938730 (bedpe file)^18^. The .hic file from experiment was downloaded and converted to .cool format using hic2cool https://github.com/4dn-dcic/hic2cool with default parameters. Contact matrices were obtained using Cooler^44^ at 5kb resolution using KR balance and log-transformed for visualisation. To quantify histogram of contact strengths for all islet loops, loop bases were extended by two 5kb HiC bins up- and downstream and the mean of the KR balance normalised matrix of this region quantified per loop.

### scRNA-seq Expression Datasets

scRNA-seq data collected over a time course of pancreatic differentiation^19^ projected onto a differentiation pseudotime was obtained from the reference’s supplementary table. We identity consistent temporal trends using Gaussian Process (GP) regression, following the approach we have previously applied^45^. Briefly, to stabilise variance we transform data log(αy+ β) where y is the gene expression for a given gene and α = 100, β = 1, then perform GP regression using Matern52 kernel and invert the transformation to report GP median and 95% confidence intervals. All GP regression was performed with GaussianProcesses.jl (https://github.com/STOR-i/GaussianProcesses.jl; https://arxiv.org/abs/1812.09064).

For scRNA-seq data in human islets^30^ accession GSE101207 we used gene counts per cell for the size healthy donors and normalised by depth per cell. In Extended Data Figures 3, 7 and 8 we show boxplots of normalised counts for each gene over healthy donors.

Finally, we obtained data on gene expression during beta-cell maturation from the Broad Single Cell portal^20^. In Extended Data Figures 3, 7 and 8 we show mean normalised counts for each gene over annotated cell types.

### Motif analysis

To assess motifs maximally disrupted by the variants, we took all motifs in the non-redundant JASPAR database^46^ and found the maximal scoring match that spanned the position of each variant for both reference and mutated sequences using https://github.com/exeter-tfs/MotifScanner.jl. Motif scores are likelihood-ratio scores of motif PWM over a background of the A,C,G,T frequencies in the hg19 genome. We normalised all motif scores, by the maximal score for each PWM and to both primary and secondary candidate motifs that may be disrupted by the variants. We considered two tiers; Tier 1 having normalised motif score ≥ 0.6, and Tier 2 having 0.45 ≤ normalised motif score < 0.45. For each tier we calculated a disruption score by subtracting the mutated sequence score from the reference sequence score and ranked these across all motifs. To report a single disrupted motif family, we grouped multiple motifs with overlapping alignments by family by removing any trailing numbers from the gene symbol (e.g. NKX2-2 → NKX2, HIC2 → HIC) and all FOX factors we grouped in the FOX family. Tier 1 disrupted motif families include NFAT, NKX2 and FOX (Figure 3e). Patient 11 has two de novo variants, the leftmost is shared with other patients and is a candidate for NFAT disruption, and the rightmost interrupts a HIC family motif (Extended Data Figure 8a). HIC family members include HIC1 and HIC2, with HIC2 the most greatly expressed in beta-cells (Extended Data Figure 8b). Interestingly, HIC2 is a transactivator of SIRT1^47^ and the loss of SIRT1 impairs glucose sensing in beta-cells in mice^48^. Tier 2 disrupted motif families include TEAD and SMAD (Extended Data Figure 8c). The TEAD family motif shares TTCA consensus with the NFAT family and is an alternative candidate to NFAT, TEAD1 is expressed in beta-cells (Extended Data Figure 8d) and plays a critical role in pancreatic progenitors^22^, however it should be noted that TEAD1 does not bind the critical region in pancreatic progenitors when the region is bound by FOXA2 (Figure 3b). Multiple members of the SMAD family are expressed in beta-cells (Extended Data Figure 8e), SMAD factors are signal transducers of TGF-beta signalling and play an important role in beta-cell development, function, and proliferation^49^.

### Statistics and Reproducibility

No statistical method was used to predetermine sample size as this study involved genetic testing in individuals with a rare monogenic disease.

**Figure 2:**
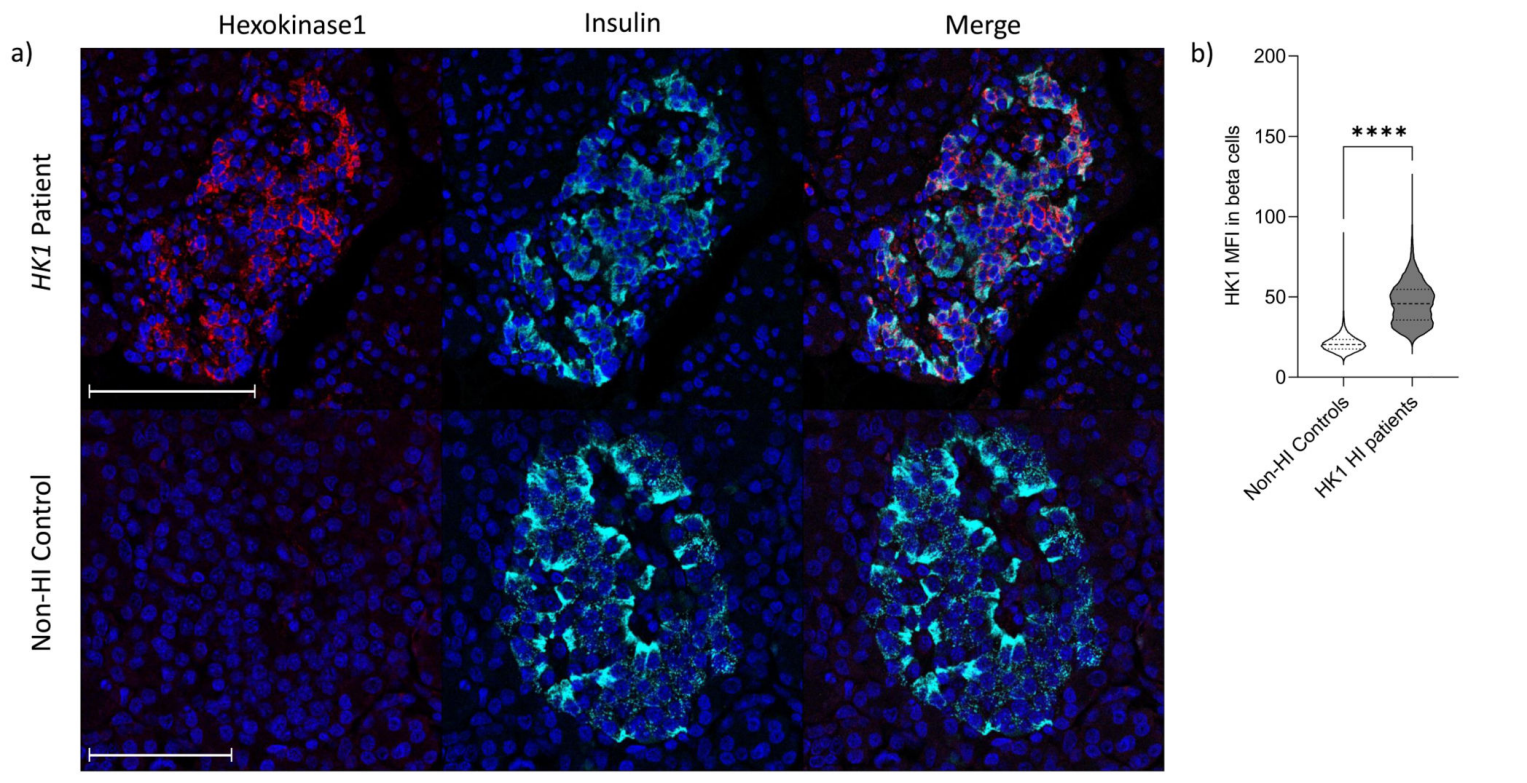
HK1 expression is present in the beta-cells of donors with *HK1*-variant CHI but not in non-hyperinsulinism (Non-HI) controls. **a)** Staining of HK1 (red); insulin (cyan) and DAPI (dark blue) in pancreatic tissue resected from patient 6 (Supplementary Table 2) with a *HK1* variant. Confocal imaging of sections demonstrates that HK1 co-localises with insulin whereas, no HK1 expression is observed in the non-HI control donor. Scale bar – 100µm. **b)** HK1 expression (Median fluorescence intensity (MFI)) is significantly increased in the *HK1* HI donors (Patient 1 and 6; n=2 donors; 17015 beta-cells), when compared to Non-HI control donors (n=2 donors; 21408 beta-cells). Two-tailed Mann-Whitney (U=590830; ****p<0.0001). All cell data within a violin plot with the median (line) and IQR (dashed lines) presented. Source data provided in Supplementary Information.

**Figure 3:**
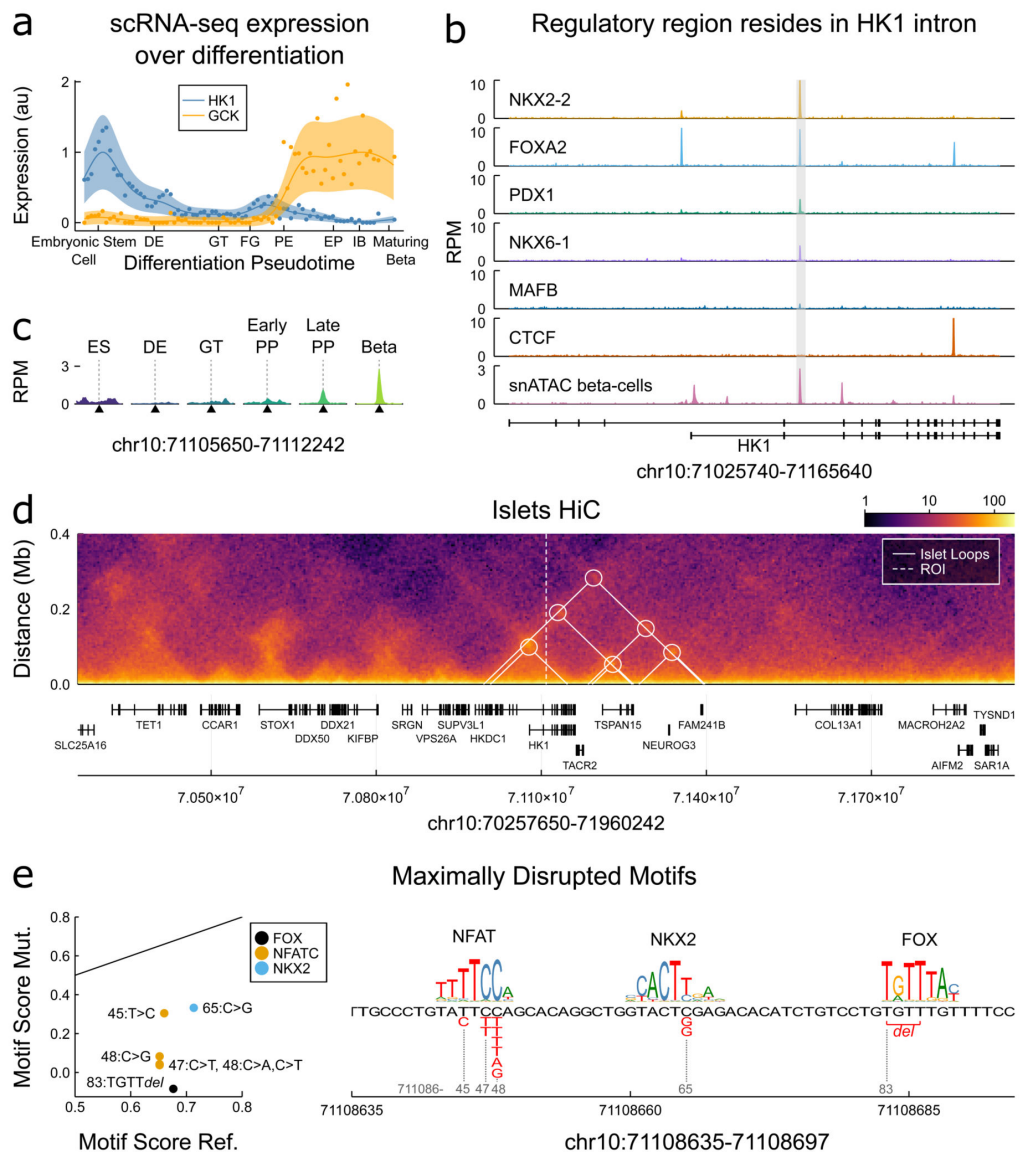
HK1 variants disrupt transcription factor binding sites within a cis-regulatory region **a)** Expression of HK1 and GCK for comparison over the course of pancreatic differentiation from embryonic stem cells to maturing beta-cells, expression data over a beta-cell differentiation pseudotime^19^, Gaussian process regression median (line) and 95% confidence interval (shaded regions)I shown. DE – definitive endoderm, GT – gut tube, FG – foregut, PE – pancreatic endoderm, EP – endocrine precursors, IB – immature beta-cells. See Extended Data Figure 3 for further HK1 expression. **b)** Transcription factor binding in human islets^16^ and chromatin accessibility from snATAC-seq in the cluster of cells assigned to beta-cells^17^ over *HK1* locus, the region containing the variants is marked as grey box. Chromatin accessibility in other endocrine cell types is provided in Extended Data Figure 6c) Chromatin accessibility during pancreatic differentiation over critical region, stages: ES – embryonic stem cell, DE – definitive endoderm, GT – gut tube, Early/Late PP – pancreatic progenitor, describe ATAC-seq from in vitro differentiation^21^ Beta cells give accessibility in snATAC-seq^17^ HiC data in human islets **d)**^18^ is contained within a well-insulated domain, heatmap gives log scale contact frequencies, white triangle and circles mark chromatin loops called in same study that span HK1, additional loops marked in Extended Data Figure 5 mark chromatin loops called in same study that span HK1, additional loops marked in . Region ± 850kb of HK1. Region ± 850kb of HK1. **e)** Transcription factor motif families disrupted by the variants, shown are those motif families maximally disrupted by each variant (red font). Notable secondary motif families given in Extended Data Figure 8 and Supplementary Table 6. Left, scatterplot of normalised motif scores for the reference versus mutated score (motif score normalised by maximal motif score), black line gives 1-1 line of equal motif. Number gives final two digits of chromatin position, e.g. 45 – 71108645. Right gives sequence logo for the three maximally disrupted families. Members of each family are expressed in beta-cells Extended Data Figure 7.

Immunofluorescence studies were performed on pancreatic samples from 6 donors. This included two individuals with *HK1*-hyperinsulinism (patients 1 and 6, Supplementary Table 2), two *ABCC8*-HI controls and two non-HI controls. Each experiment was performed once due to limited sample availability. Representative data from these imaging studies are shown for one of each donor type in Figure 2a and Extended Data Figure 4a-b.

No data were excluded from any of the experiments described.

## Extended Data

**Extended Data Figure 1:**
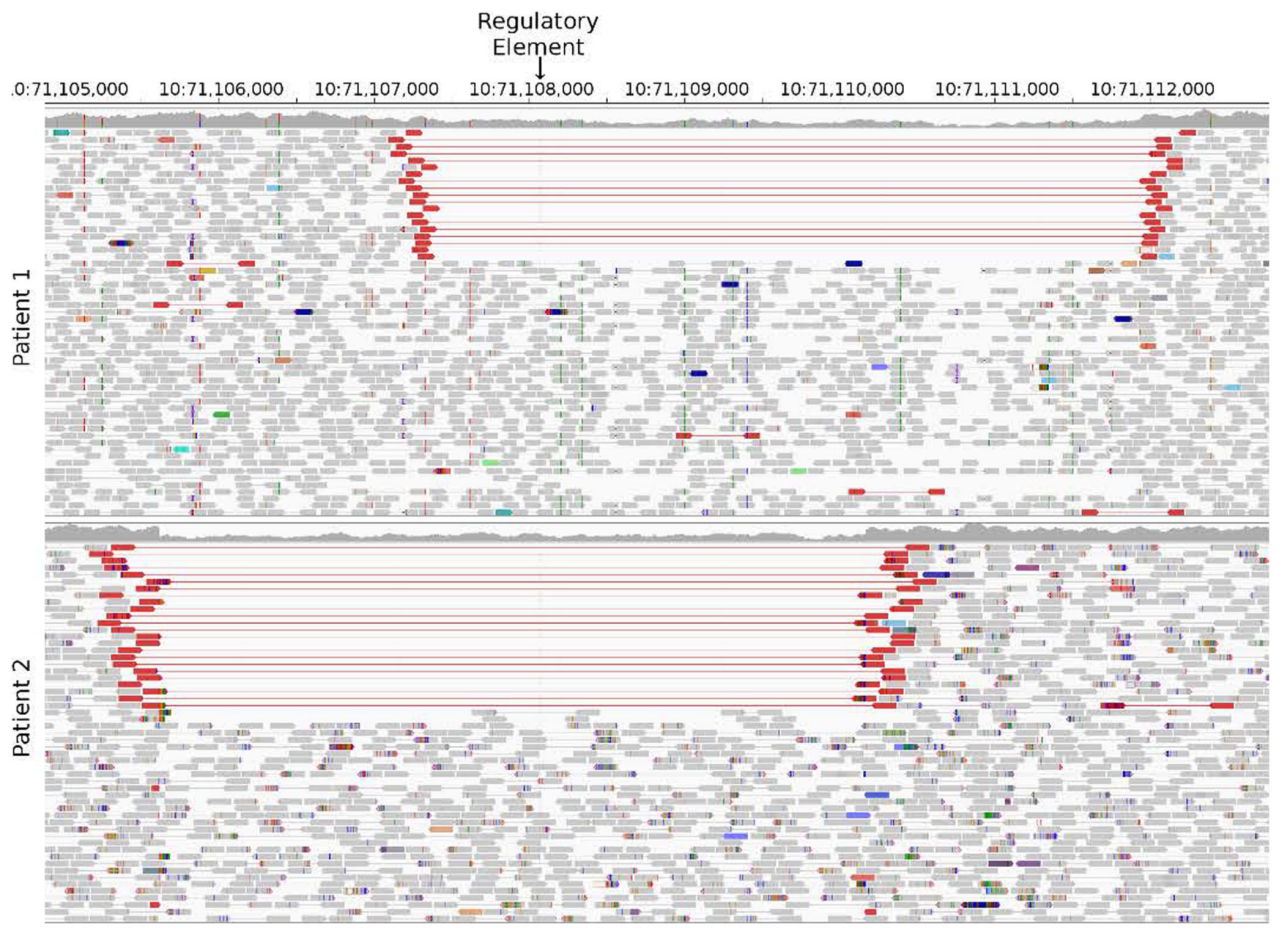
Integrative Genomics Viewer (IGV) screenshot of the ~4.5Kb deleted regions encompassing the regulatory element in intron 2 of *HK1* in patients 1 and 2.1. Both deletions are heterozygous, so reads are present in the deleted regions from the other haplotype, although the read depth is reduced. Reads highlighted in red have a long insert size. Both features are consistent with a deletion. The deleted region in patient 2.1 has detectable break points, as shown by the discontinuity in read depth and the reads clipped at the deletion boundary. In patient 1, the breakpoints do not result in read depth discontinuity or clipped reads as the deletion occurs between two matching repeating sequences.

**Extended Data Figure 2:**
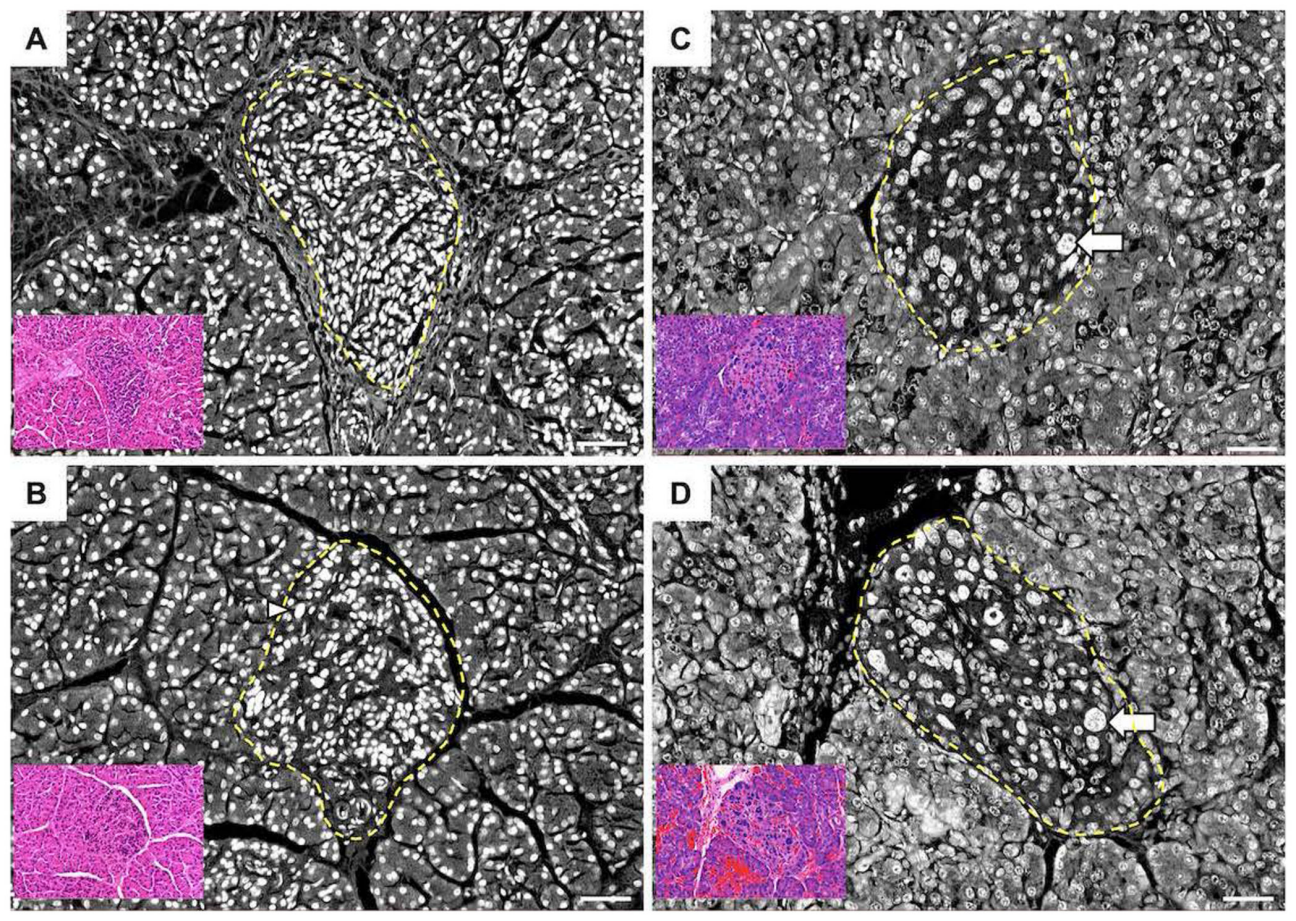
Histological appearance of islets from *HK1*-CHI and *ABCC8*-HI tissue. Panels **A** and **B** illustrate islets from two *HK1* CHI patients (patients 1 and 6 Supplemental Table 2) in comparison to age-matched tissue from *ABCC8* CHI patients with diffuse pancreatic disease **(C and D).** The yellow dashed line delineates a single islet in each sample. Nuclear enlargement (B, arrowhead) and nucleomegaly (C, D arrows) was virtually undetected in control islets (n=12/72,145 islet cells) but found in 0.8% of HK1 islets (n=374/46,885 cells) and 4.9% of *ABCC8* islet cells (n=70,929). Inserts illustrate the corresponding haematoxylin and eosin images. Scale bar 50 µm

**Extended Data Figure 3:**
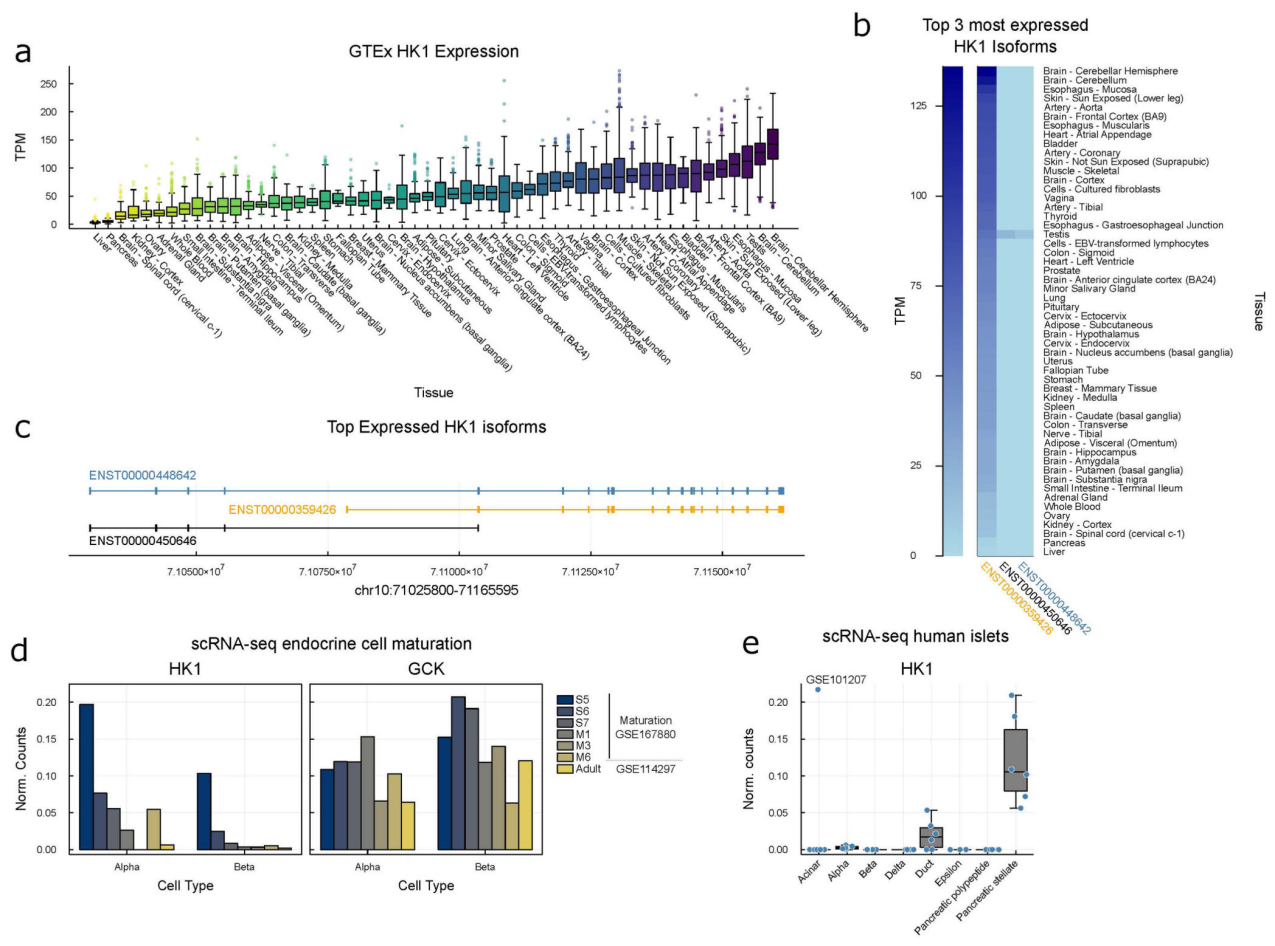
HK1 expression in GTEx **a)** Boxplots describing gene level HK1 expression in Genotype-Tissue Expression GTEx project ordered by expression with least expression in liver and pancreas. Data shows sum of GTEx v8 isoform level data calculated with RSEM. The GTEx Project was supported by the Common Fund of the Office of the Director of the National Institutes of Health, and by NCI, NHGRI, NHLBI, NIDA, NIMH, and NINDS. The data used for the analyses described in this manuscript were obtained from the GTEx Portal on 08/26/21. **b)** Isoform level HK1 expression in GTEX tissues as **a)**, top 3 most expressed isoforms are given. Long HK1 isoform only expressed in testis, short isoform ubiquitously expressed across all tissues. **c)** Transcript models of top three HK1 isoforms given in b). **d)** Expression of HK1 and GCK for comparison in scRNA-seq across alpha and beta-cell maturation, all data quantified by (GSE167880)^20^, which quantified their own data over maturation and adult endocrine cells (GSE114297)^31^. **e)** HK1 expression in human islet cell types by scRNA-seq (GSE101207)^30^. Data points from independent human donors, the central lines in the boxplots correspond to the median, boxes span from the first to the third quartiles, and whiskers extend to the furthest data point within 1.5xIQR from the boxes. HK1 expression only in duct and pancreatic stellate cells, expression absent in endocrine cell types.

**Extended Data Figure 4:**
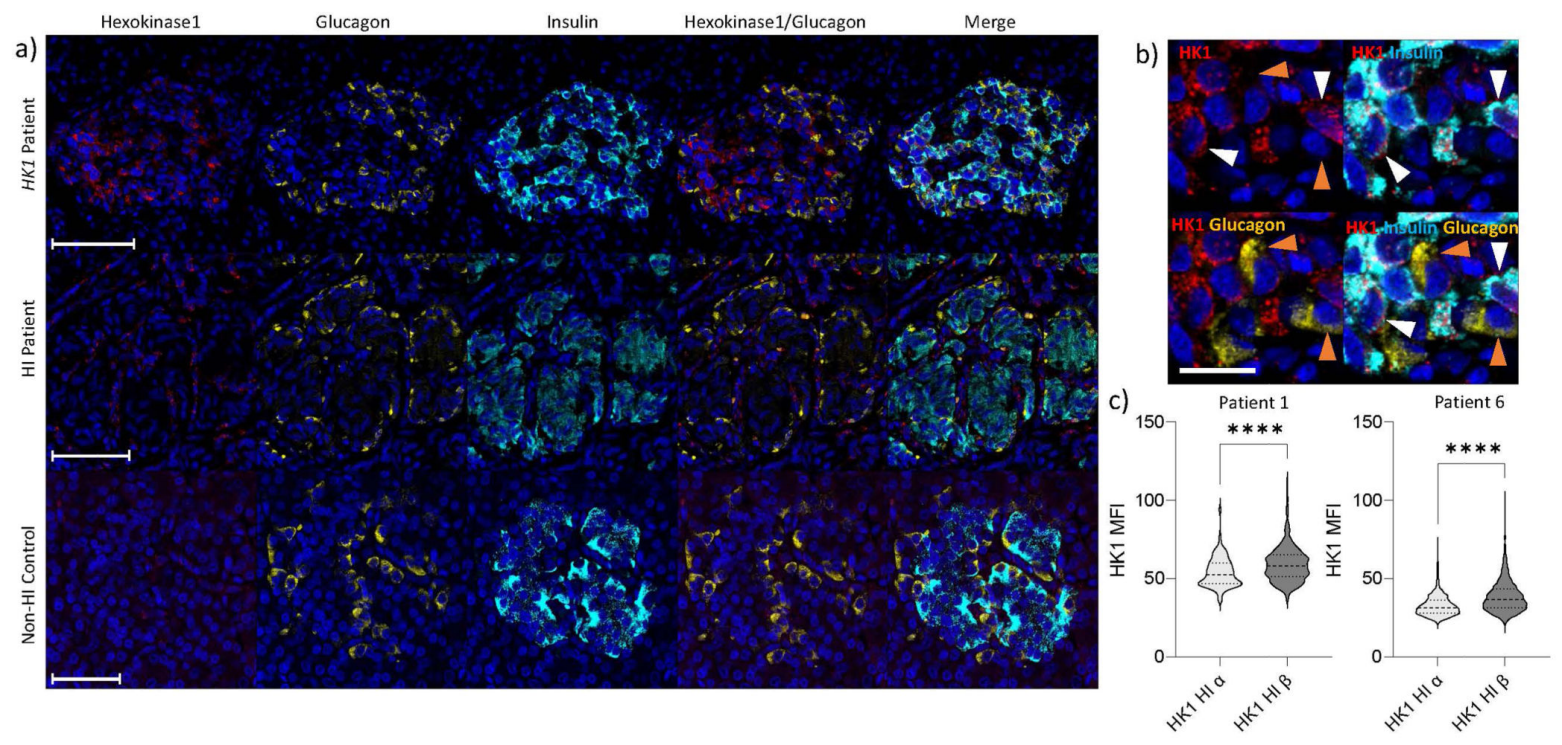
HK1 expression is present in the beta-cells of donors with *HK1*-CHI, but not *ABCC8*-Hyperinsulinism or Non-HI controls. **a)** Confocal imaging of HK1 (red); glucagon (yellow); insulin (cyan) and DAPI (dark blue) in pancreatic tissue resected from patient 6 (Supplementary Table 2) with a *HK1* variant. HK1 co-localises with insulin, but not with glucagon. Staining in tissue resected from individuals with *ABCC8*-Hyperinsulinism (HI-control) and non-HI control, showed no expression of HK1 in either alpha (glucagon-positive) or beta (insulin-positive cells). Scale bar – 100 µm. **b)** Enlarged image of a portion of the islet shown in (a) from an individual with *HK1*-HI (Patient 6). This image confirms the expression of HK1 specifically in beta cells (white arrows) and absence of HK1 in alpha cells (orange arrows). Scale bar – 20 µm **c)** HK1 expression (Median fluorescence intensity (MFI)) is higher in the *HK1*-HI donor beta-cells within HK1 positive islets, when compared to alpha cells (Patient 1 - beta-cells (n=1580); alpha-cells (n=161); and Patient 6 - beta-cells (n=2284); alpha-cells (n=9321)). Two-tailed Mann-Whitney (****p<0.0001). This suggests the impact of the *HK1* variant is beta-cell specific. Violin plots shown with the median (dashed line) and IQR (dotted lines) presented. Source data provided in Supplementary Information.

**Extended Data Figure 5:**
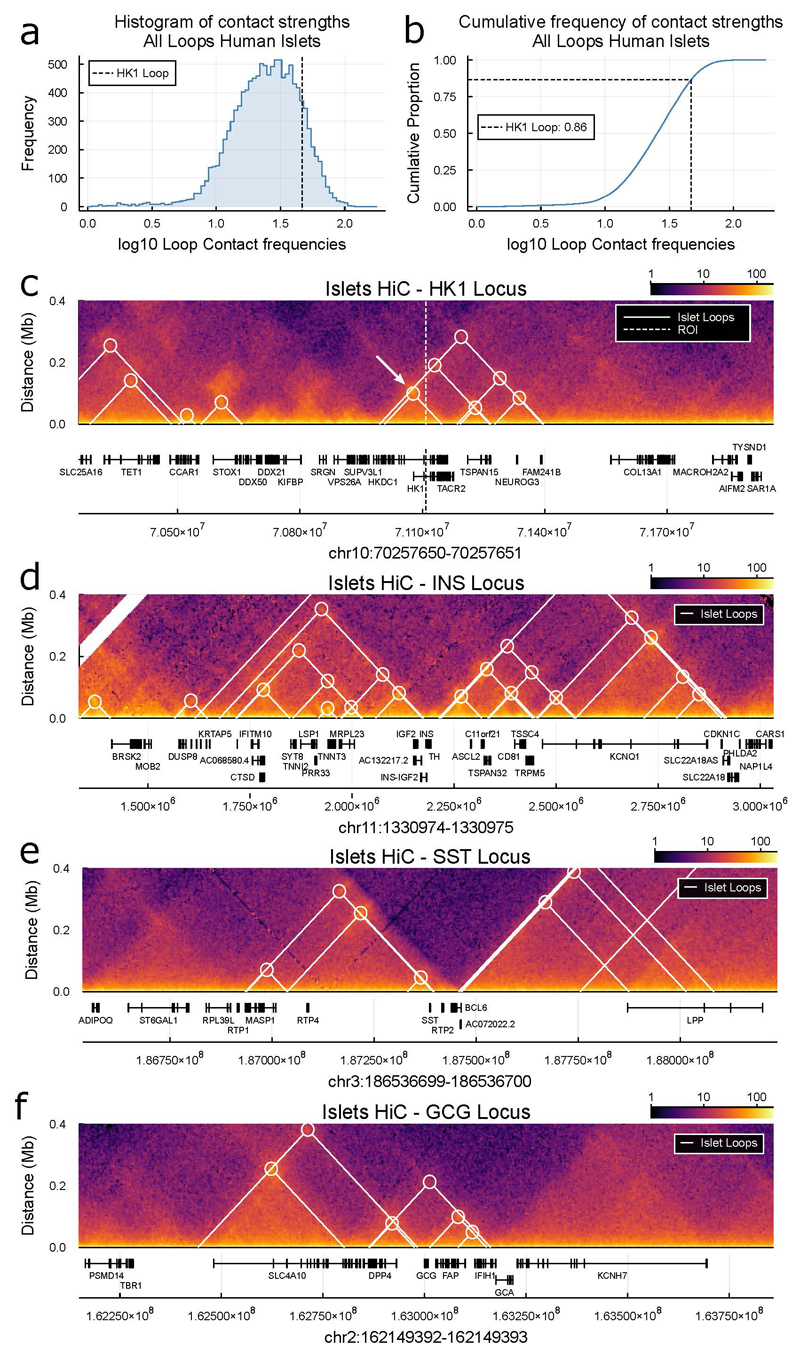
Human Islet chromatin conformation at loci spanning HK1 and secreted endocrine genes. **a)** Histogram of contact strengths of loops in human islet HiC data^18^, data shown for all loops called in that study. Vertical line marks the strength of strongest and smallest loop spanning HK1 gene (marked with white arrow in **c)**). **b)** Cumulative frequency plot of contacts shown in **a)**, vertical line marks HK1 loop (white arrow in **c)**), the HK1 loop is in the 86^th^ percentile of all islet HiC loop strengths. **c**) Heatmap of HiC data shown in **a)** for region ± 850 kb of HK1 as Figure 3d, now with additional loops within region marked. White arrow gives principal loop spanning HK1 gene marked in **a, b). d,e,f)** HiC data and loops for endocrine cell secreted genes **d)** INS, **e)** SST, and **f)** GCG, shown at same resolution and scale as HK1 locus **c)** for context.

**Extended Data Figure 6:**
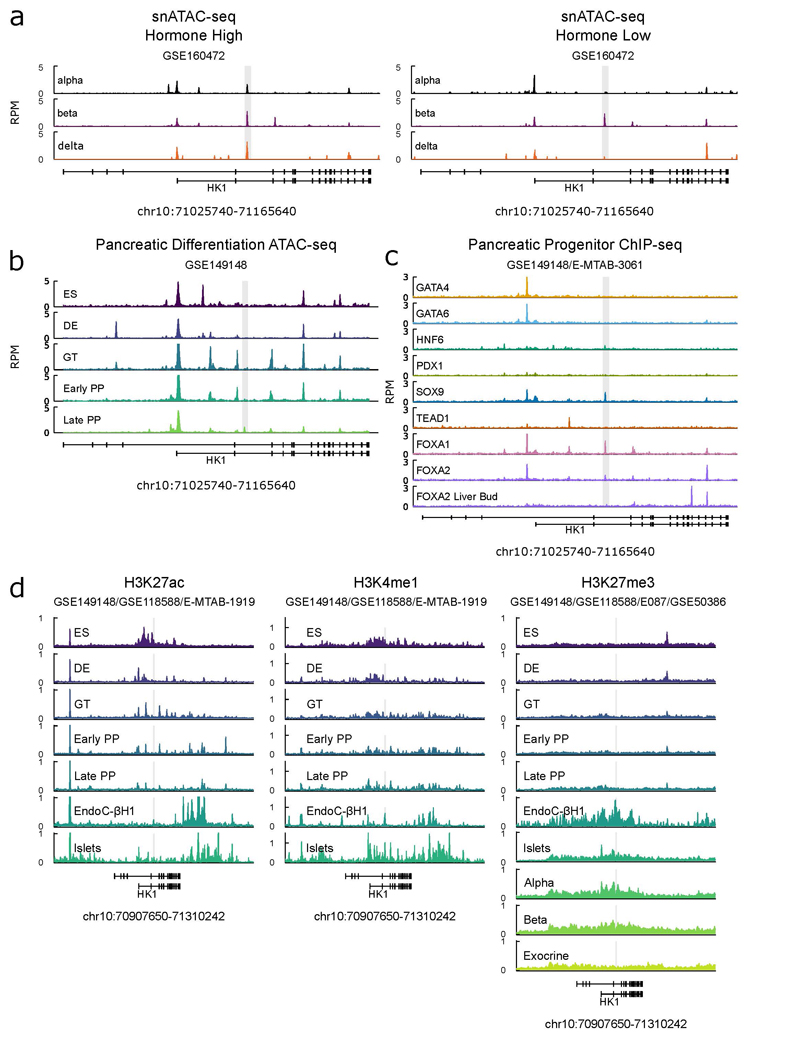
Chromatin accessibility, Transcription Factor binding and Epigenetic modifications at the *HK1* locus over pancreatic differentiation **a)** snATAC-seq data in human islets showing mean chromatin accessibility over nuclei assigned to alpha, beta and delta cell clusters in hormone high and hormone low conditions (GSE160472)^17^. Critical region containing variants encompassed within grey box. Alpha, beta and delta cells each show accessibility in hormone high state, whereas only beta-cells retain accessibility in hormone low cells. Hormone high and low states characterised by level of accessibility over endocrine cell secreted hormone promoter, alpha – GCG, beta – INS, delta – SST. Data show in reads per million (RPM). **b)** Chromatin accessibility of pancreatic differentiation assessed by ATAC-seq (GSE149148)^21^, wider locus view of same data shown in Figure 3c. Critical region containing variants encompassed within grey box. Stages: ES – embryonic stem cell, DE – definitive endoderm, Early/Late PP – pancreatic progenitor. **c)** Transcription factor binding in pancreatic progenitors (GSE149148)^21^ and in Carnegie Stage 16-18 liver bud for FOXA2 (E-MTAB-3061)^22^, data shown as a) and b). Liver bud data reveals that whilst FOXA2 binds critical region in pancreatic progenitors and human islets (Figure 3b), it does not bind in liver bud, suggesting that critical region encodes pancreas specific regulation of *HK1*. **d)** Epigenetic modifications over *HK1* locus, shown are active marks H3K27ac and H3K4me1 and repressive mark H3K27me3 over in vitro pancreatic cell differentiation (GSE149148)^21^ stages as in **b)**; in vitro beta-cell line EndoC-BH1 (GSE118588)^25^ and human islets H3K27ac and H3K4me1 (E-MTAB-1919)^16^ and H3K27me3 (E087 – roadmap epienomics^23^); cell-sorted pancreatic alpha, beta and exocrine cells (GSE50386)^24^. Grey box encompasses critical region containing variants. Data reveals that H3K27ac broadly marks short isoform promoter (**Supplemental Figure 3**) at ES cells stage is reduced by DE stage, whilst focal mark over regulatory region is present in EndoC-BH1 and human islets. Similarly, H3K4me1 shows string focal enrichment over regulatory region in EndoC-BH1 and islets. Finally, repressive mark H3K27me3 is absent over the locus over the course of pancreatic cell differentiation and is present in a broad domain in adult islets, EndoC-BH1 cells, beta, cells and to reduced extent in exocrine cells.

**Extended Data Figure 7:**
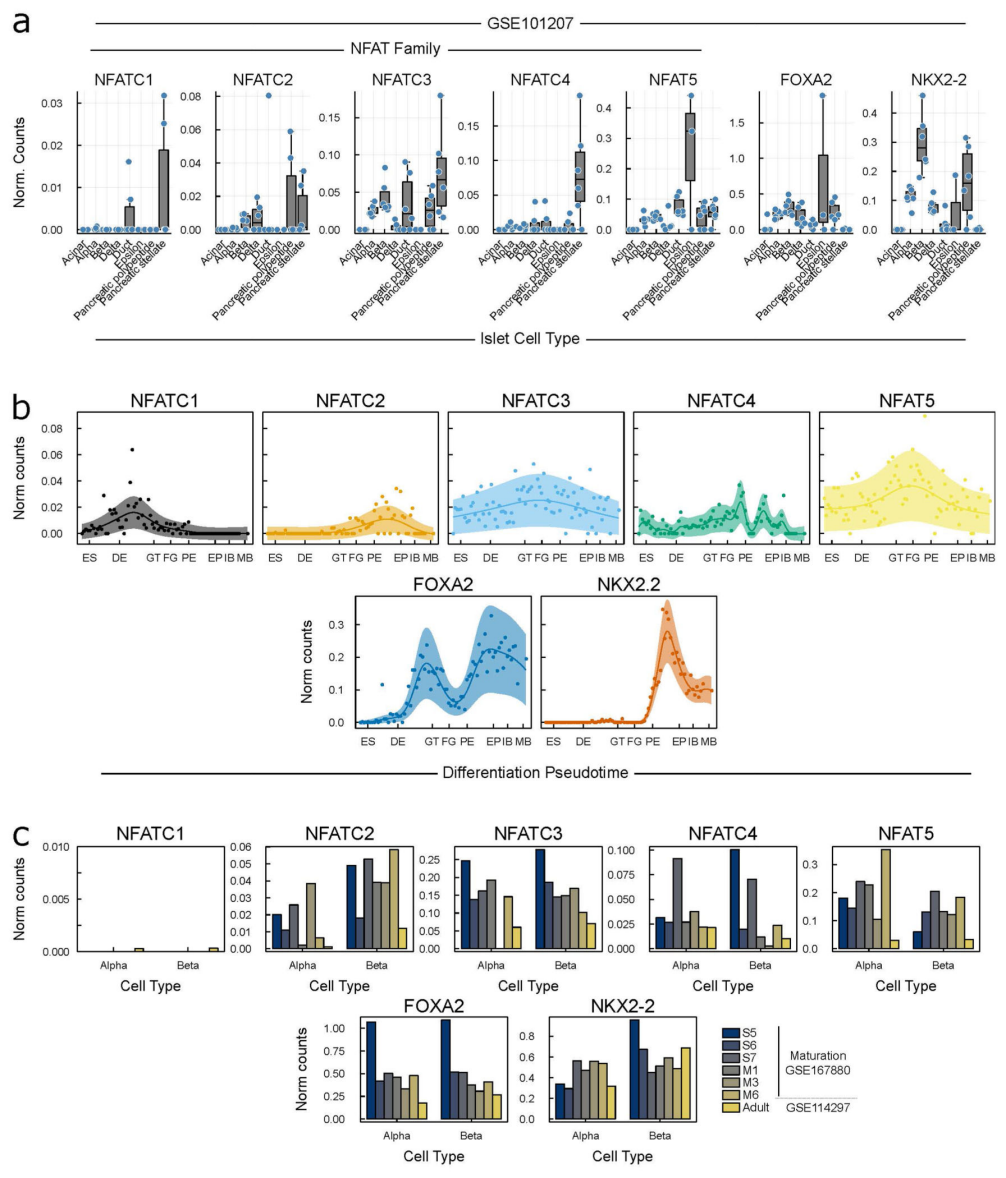
Expression of family members of disrupted transcription factor binding motifs in critical region NFAT family, NKX2-2 and FOXA2: **a)** In single-cell RNA-seq in human islets (GSE101207)^30^, boxplots of mean normalised counts from independent islet donors in cells assigned to islet cell type clusters (data shown as in **Supplemental Figure 3e**). Data points from independent human donors, the central lines correspond to the median, boxes span from the first to the third quartiles, and whiskers extend to the furthest data point within 1.5xIQR from the boxes. **b)** Over the course of pancreatic differentiation from embryonic stem cells to maturing beta-cells, expression data over a beta-cell differentiation psuedotime^19^, Gaussian process regression median and 95% CI shown. ES – embryonic stem cell, DE – definitive endoderm, GT – gut tube, FG – foregut, PE – pancreatic endoderm, EP – endocrine precursors, IB – immature beta-cells, MB – maturing beta-cells. **c)** scRNA-seq across alpha and beta-cell maturation, all data quantified by (GSE167880)^20^, which quantified their own data over maturation and adult endocrine cells (GSE114297)^31^.

**Extended Data Figure 8:**
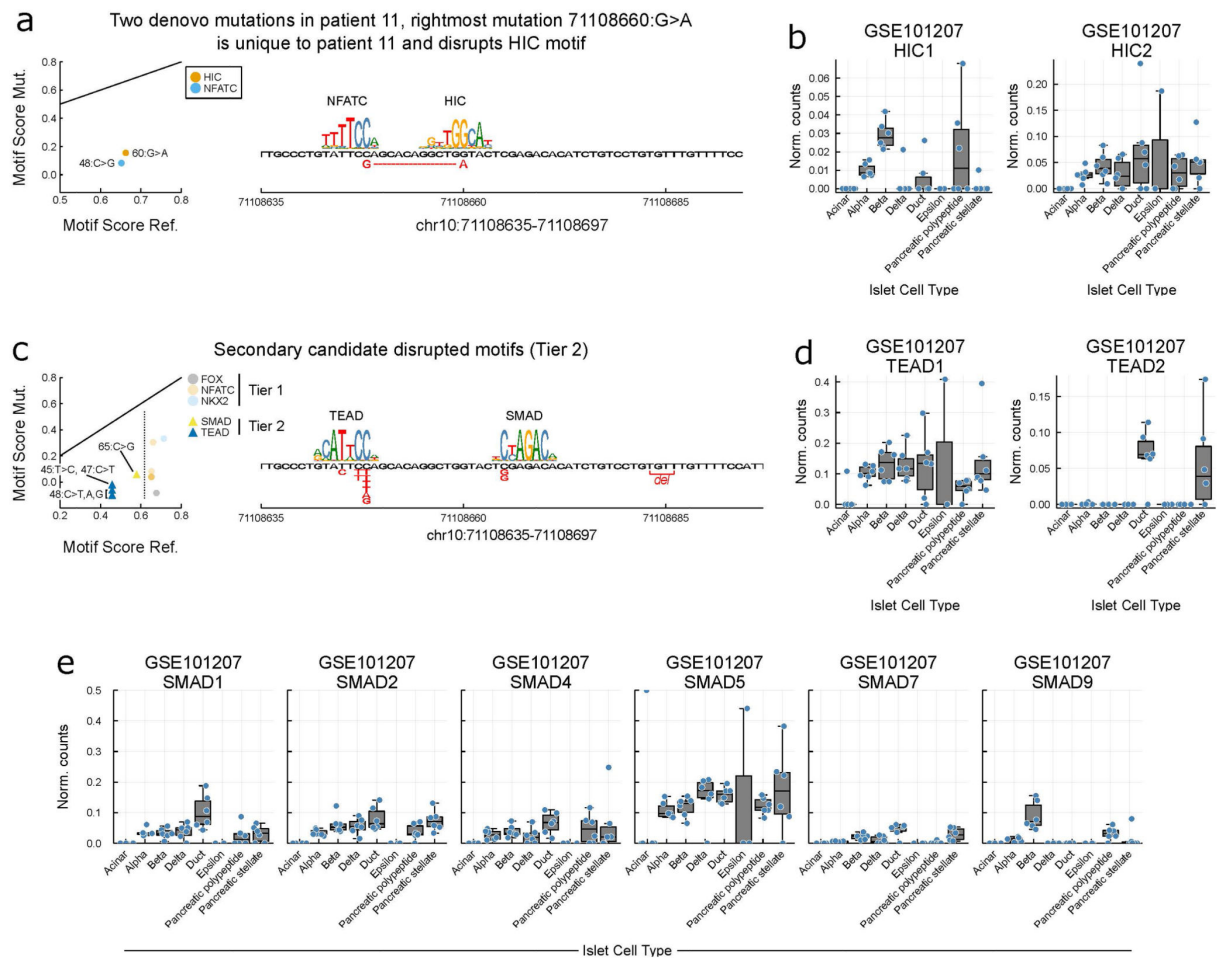
Additional transcription factor motifs disrupted by variants **a-b)** Sanger sequencing identified 2 *in cis de novo* variants 12 base pairs apart (g.71,108,648C>G and g.71,108,660G>A) in patient 11. g.71,108,648 is affected by 3 different *de novo* substitutions in 6 individuals and resides within a predicted NFATC binding motif. Two further *de novo* variants identified in 3 patients are also predicted to impact on NFATC binding (Figure 3). In contrast, further variants affecting g.71,108,660 have not been identified, this suggests that the g.71,108,648C>G is causative of the disease in this patient. Nevertheless, it is of note that g.71,108,660G>A is predicted to affect a HIC family motif **a)** and human islet scRNA-seq (GSE101207)^30^ determines HIC2 is expressed in beta-cells **b)** Interestingly, HIC2 is a transactivator of SIRT1^47^ and the loss of SIRT1 impairs glucose sensing in beta-cells in mice^48^. **c-e)** Tier 2, secondary candidate motifs for disruption have lower normalised motif scores than Tier 1 matches. **c)** Two additional motif families are implicated TEAD and SMAD. TEAD shares partial consensus with NFAT (TTCCA) and is alternative candidate to NFAT. TEAD1 but not TEAD2 is expressed in beta-cells **e**) and TEAD1 plays a critical role in pancreatic progenitors^22^, however it should be noted that TEAD1 does not bind the critical region in pancreatic progenitors when the region is bound by FOXA2 (Figure 3b). The SMAD family are signal transducers of TGF-beta signalling and play an important role in beta-cell development, function, and proliferation^49^. For all boxplots, in panels (b, d, e) central lines correspond to the median, boxes span from the first to the third quartiles, and whiskers extend to the furthest data point within 1.5xIQR from the boxes.

## Supplementary Material

Reporting summary

Source Data Extended Data Figure 4

Source Data Figure 2

Supplementary table 6

Supplementary tables

## Figures and Tables

**Figure 1:**
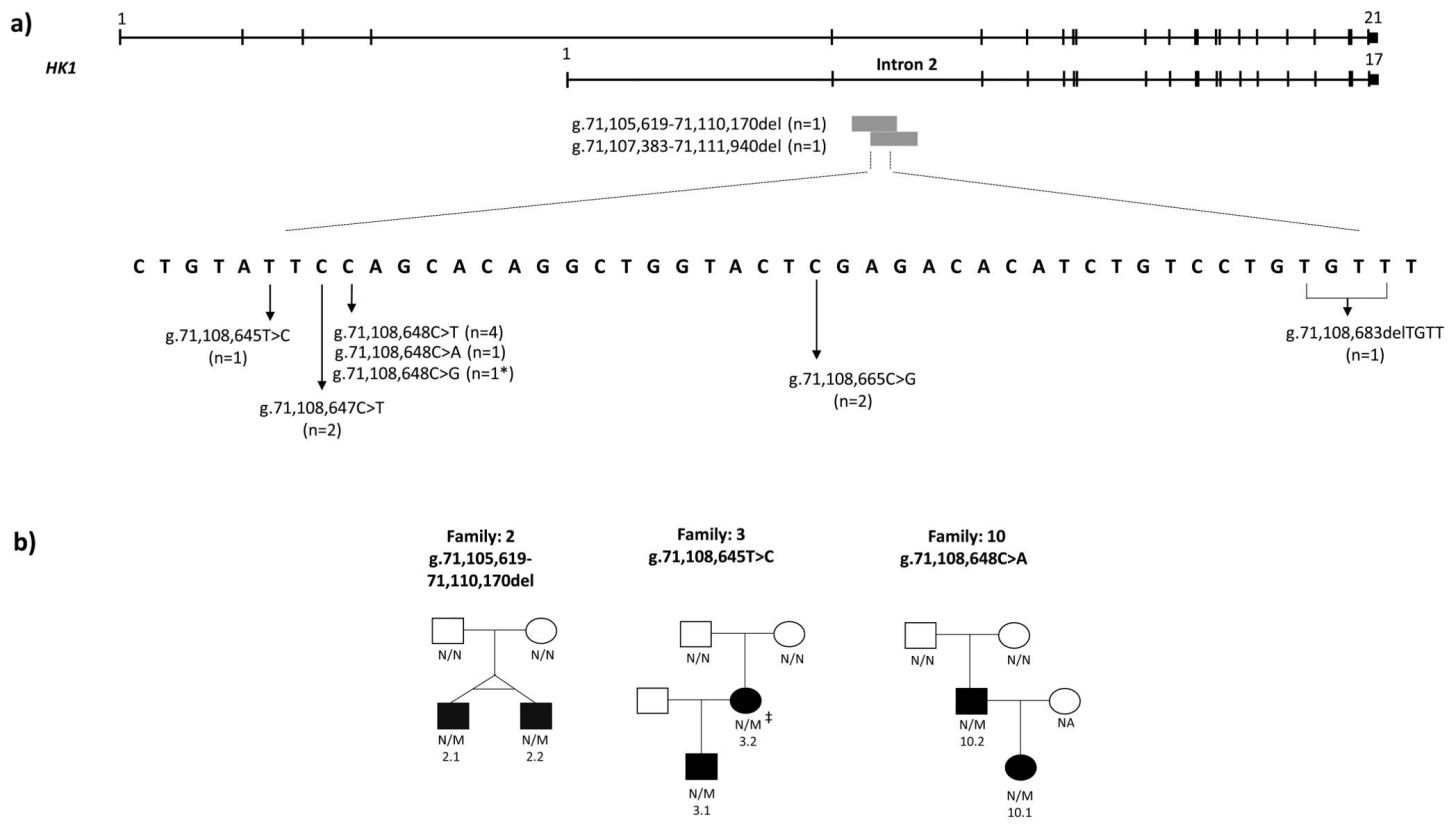
Schematic representation of the *HK1* gene and the identified variants. **a)** Schematic representation of the *HK1* gene (GRCh37/hg19 chr10:71,029,756-71,161,637). The full-length testis-specific isoform (ENST00000448642) and the shorter ubiquitously expressed isoform (ENST00000359426) are depicted. The positions of the two ~4.5Kb deletions within intron 2 of the shorter isoform and the 7 different heterozygous single nucleotide variants and indels within the minimal deleted region identified in 14 probands are shown. The number of probands with each variant is provided. An asterisk (*) denotes a single proband with two *de novo* variants (see Extended Data Figure 8). The variant positions are given according to the GRCh37/hg19 genomic coordinates. **b)** Partial pedigrees showing inherited *HK1* variants in 3 families. Filled symbols represent individuals with congenital hyperinsulinism. (‡) Haplotype analysis confirmed that the *HK1* variant had arisen *de novo* in patient 3.2. M, *HK1* variant; N, no variant; NA, DNA not available. Pedigrees for the 11 probands with *de novo HK1* variants are not shown.

## Data Availability

All non-clinical data analysed during this study are included in this published article (and its supplementary information files). Clinical and genotype data can be used to identify individuals and is therefore available only through collaboration to experienced teams working on approved studies examining the mechanisms, cause, diagnosis and treatment of diabetes and other beta-cell disorders. Requests for collaboration will be considered by a steering committee following an application to the Genetic Beta Cell Research Bank (https://www.diabetesgenes.org/current-research/genetic-beta-cell-research-bank/). Contact by email should be directed to Prof Sarah Flanagan (S.Flanagan@exeter.ac.uk). All requests for access to data will be responded to within 14 days. Accession codes and DOI numbers for all ChIP-seq, ATAC-seq, RNA-seq and scRNA-seq datasets are provided in Supplementary Table 5. We used the Genome Reference Consortium Human Build 37 (GRCh37) to annotate genetic data. Accession number: GCF_000001405.13. Details of this assembly are provided at: https://www.ncbi.nlm.nih.gov/assembly/GCF_000001405.13/. Source data for Figure 2b and Extended Figure 4c are provided in the Additional Supplementary Files. For Figure 3 and Extended Data Figures 3, 5, 6, 7, 8 all source data is contained in https://github.com/owensnick/HK1FigureNotebook.jl and DOI:10.5281/zenodo.6815326.

## Code Availability

The code used in this study can be freely downloaded from:

https://github.com/rdemolgen/SavvySuite

https://github.com/owensnick/HK1FigureNotebook.jl

https://github.com/owensnick/GenomeFragments.jl

https://github.com/kjgaulton/pipelines/tree/master/islet_snATAC_pipeline

https://github.com/4dn-dcic/hic2cool

https://github.com/STOR-i/GaussianProcesses.jl

https://github.com/exeter-tfs/MotifScanner.jl

https://qupath.github.io
