# Supplementary material for: Non-coding variants disrupting a tissue-specific regulatory element in HK1 cause congenital hyperinsulinism
Source: Nat Genet. Author manuscript; Available in PMC 2023 Jan 9. (PMC7614032; doi:10.1038/s41588-022-01204-x)
Supplement: Supplementary tables [file EMS154463-supplement-Supplementary_tables.docx]

**Supplementary Information**

Non-coding variants disrupting a tissue-specific regulatory element in *HK1* cause congenital hyperinsulinism

**Authors**

Matthew N. Wakeling^1^*, Nick D. L. Owens^1^*, Jessica R. Hopkinson^1^, Matthew B. Johnson^1^, Jayne A.L. Houghton^2^, Antonia Dastamani^3^, Christine S. Flaxman^1^, Rebecca C. Wyatt^1^, Thomas I. Hewat^1^, Jasmin J. Hopkins^1^, Thomas W. Laver^1^, Rachel van Heugten^2^, Michael N. Weedon^1^, Elisa De Franco^1^, Kashyap A. Patel^1^, Sian Ellard^2^, Noel G. Morgan^1^, Edmund Cheesman^4^, Indraneel Banerjee^5,6^, Andrew T. Hattersley^1^, Mark J. Dunne^6^, International Congenital Hyperinsulinism Consortium^¥^, Sarah J. Richardson^1^ and, Sarah E. Flanagan^1^

**Affiliations**

1. Institute of Biomedical and Clinical Science, University of Exeter Medical School, UK
2. Exeter Genomics Laboratory, Royal Devon and Exeter NHS Foundation Trust, Exeter, UK
3. Endocrinology Department, Great Ormond Street Hospital for Children, London, UK
4. Department of Paediatric Pathology, Royal Manchester Children’s Hospital, Oxford Road, Manchester, UK
5. Department of Paediatric Endocrinology, Royal Manchester Children’s Hospital, Oxford Road, Manchester, UK
6. Faculty of Biology, Medicine and Health, The University of Manchester, Oxford Road, Manchester, UK

* The authors contributed equally to this work.

¥ A list of the authors and their affiliations appears at the end of the paper.

**Supplementary Table 1:** Summarised clinical characteristics for 162 probands diagnosed with congenital hyperinsulinism screened for variants in the *HK1* regulatory region by whole-genome sequencing (n=135) or Sanger sequencing and ddPCR (n=27). Median values (IQR) provided**.**

|  | **Summary data** |
| --- | --- |
| **Female sex** | 80 (49.1%) |
| **Birth weight SDS** | 0.10  (-1.16 – 1.14) |
| **Age at onset (days)** | 56  (7 – 168) |
| **Blood glucose at presentation (mmol/l)** | 1.6  (1.2 – 2.1) |
| **Insulin at presentation (pmol/l)** | 48  (25 – 100) |
| **Pancreatectomy performed** | 34 (20.8%) |

**Supplementary Table 2:** Clinical characteristics of 17 individuals with a *HK1* variant. The variant positions are given according to the GRCh37/hg19 genomic coordinates. The level of mosaicism in leukocyte DNA is estimated by looking at the proportion of reads with the variant at that position.

|  | **Patient 1** | **Patient 2.1** | **Patient 2.2** | **Patient 3.1** | **Patient 3.2** | **Patient 4** | **Patient 5** | **Patient 6** | **Patient 7** |
| --- | --- | --- | --- | --- | --- | --- | --- | --- | --- |
| ***HK1* variant (inheritance)** | g.71,107,383 - 71,111,940del (*de novo*) | g.71,105,619-71,110,170del (*de novo*) | g.71,105,619-71,110,170del (*de novo*) | g.71,108,645T>C  (dominant) | g.71,108,645T>C  (*de novo*) | g.71,108,647C>T  (*de novo*) | g.71,108,647 C>T (*de novo*) | g.71,108,648C>T  (*de novo*) | g.71,108,648 C>T  (*de novo*) |
| **Relationship to proband** | - | - | Twin of patient 2.1 | - | Mother of patient 3.1 | - | - | - | - |
| **Gender** | Female | Male | Male | Male | Female | Male | Female | Female | Female |
| **Ethnicity** | Caucasian | NZ Mãori | NZ Mãori | Caucasian | Caucasian | Caucasian | Caucasian | Caucasian | Unknown |
| **Current Age (yrs)** | 14 | 12 | 12 | 6 | 42 | 11 | 4 | 9 | 4 |
| **Birth weight (SDS)** | 1.80 | 1.95 | 0.86 | 0.78 | -0.12 | 2.22 | 0.18 | 2.03 | 2.55 |
| **Age at onset of hyperinsulinism** | Birth | Birth | Birth | 22 weeks | 13 weeks | Birth | Birth | 2 weeks | 0.42 weeks |
| **Blood glucose at diagnosis (mmol/L) [paired insulin (pmol/L)]** | 1.2 (77) | 1.1 (1771) | 0.8 (>452) | 0.8 (13) | 2.3 (11) | 1.4 (192) | 0.8 (315) | 1.7 (1240) | 2.2 (151) |
| **Current treatment (dose)** | Diazoxide (6.5mg/kg/d) | Diazoxide (10.1mg/kg/d) Cornstarch | Diazoxide (11.4mg/kg/d)  Cornstarch | Diazoxide (5mg/kg/d) | None  Diazoxide  (10mg/kg/d)  until 7 years | Diazoxide (11.5mg/kg/d) | Diazoxide (11mg/kg/d)  Lanreotide  (dose unknown) | Insulin | Diazoxide (15mg/kg/d) |
| **Pancreatic resection (age)** | Yes (23 months) | Yes (2 yrs 8 months) | Yes (2 yrs 8 months) | No | No | Yes (2.1 yrs and 2.8 yrs) | No | Yes (6 months) | No |
| **Additional Features** | Active duodenitis, exocrine insufficiency | None | None | None | None | None | None | None | None |

|  | **Patient 8** | **Patient 9** | **Patient 10.1** | **Patient 10.2** | **Patient 11** | **Patient 12** | **Patient 13** | **Patient 14** |
| --- | --- | --- | --- | --- | --- | --- | --- | --- |
| ***HK1* variant (inheritance)** | g.71,108,648C>T  (*de novo*) | g.71,108,648C>T  (*de novo*; mosaic 17%) | g.71,108,648C>A (dominant) | g.71,108,648C>A  (*de novo*) | g.71,108,648  C>G and  g.71,108,660  G>A  (both *de novo*) | g.71,108,665C>G  (*de novo*) | g.71,108,665C>G  (*de novo*) | g.71,108,683delTGTT (*de novo;* mosaic 30%) |
| **Relationship to proband** | - | - | - | Father of patient 9.1 | - | - | - | - |
| **Gender** | Female | Female | Female | Male | Female | Male | Male | Male |
| **Ethnicity** | Sephardic Jew | Caucasian | Caucasian | Caucasian | Black African | South Asian (Punjabi) | Ashkenazi Jew | Pacific Islander |
| **Current Age (yrs)** | 9 | 4 | 9 | Unknown | Deceased aged 2.5 months (sepsis) | 5 | 18 | 6 |
| **Birth weight (SDS)** | -1.27 | 0.31 | 1.38 | Unknown | 0.09 | -4.16 | 0.10 | 0.43 |
| **Age at onset of hyperinsulinism** | Birth | Birth | Birth | Unknown | 2 weeks | 12 weeks | 0.28 wks (resolved) 30 wks (relapse) | Birth |
| **Blood glucose [mmol/L] and (paired insulin [pmol/L]) at presentation** | 2.7 (118) | 2.1 (336) | Unknown | Unknown | 1.6 (236) | 1.6 (60) | 1.4 (112) | 2.0 (163) |
| **Current treatment (dose)** | Diazoxide  (2.6 mg/kg/d)  Octreotide  (16.2 mcg/kg/d) | Diazoxide (10.32mg/Kg/d)  Octreotide LAR (20mcg once monthly) | Unknown | Unknown | - | Diazoxide  (15mg/Kg/d) | Octreotide LAR | Diazoxide (13 mg/kg/d)  Octreotide LAR (30mg 3 weekly) |
| **Pancreatic surgery (age)** | No | No | No | No | Yes (2 months) | No | No | No |
| **Additional Features** | Horse-shoe kidney, Asthma, Aberrant left subclavian artery- operated | None | None | None | Microcephaly, hepatoslenomegaly | None | None | None |

**Supplementary Table 3:** Summarised clinical characteristics for 17 individuals from 14 families with *HK1* variants. Median values (IQR) provided**.**

|  | **Summary data** |
| --- | --- |
| **Sex (female)** | 9 (53%) |
| **Birth weight (SDS)** | 0.61  (0.10-1.84) |
| **Consanguinity** | 3 (18%) |
| **Age at onset** | Birth  (0-14 days) |
| **Blood glucose at presentation (mmol/l)** | 1.6  (1.2-2.1) |
| **Insulin during hypoglycaemia (pmol/l)** | 163  (95-326) |
| **Pancreatic resection performed** | 5/17 (29%) |

**Supplementary Table 4**: Primer sequences for **a)** ddPCR to confirm the deletions identified by whole-genome sequencing in patients 1, 2.1 and 2.2 **b)** ddPCR to screen for deletions in the replication cohort within the 151bp region harbouring *de novo* single nucleotide variants **c)** Sanger sequencing of the *HK1* element

**a)**

| Primer | Sequence (5’ – 3’) | Region amplified (Hg19) |
| --- | --- | --- |
| HK1_A_Forward | tccctgcagtaatcagaatctgg | Chr10:71105908-71106051 |
| HK1_A_Reverse | agcgccaatttatccctccg |  |
| HK1_B_Forward | agggtctgcgtggtctttatg | Chr10:71107602-71107732 |
| HK1_B_Reverse | ggaaacccaaaatacttcacccc |  |
| HK1_C_Forward | ctcttacagtggcagggacg | Chr10:71109774-71109906 |
| HK1_C_Reverse | aagccaatcacagccctctc |  |
| HK1_D_Forward | gtgattggtacagaaagactcagtg | Chr10:71111625-71111726 |
| HK1_D_Reverse | cagtatgaagtagctcctcgcag |  |

**b)**

| Primer | Sequence (5’-3’) | Region amplified (Hg19) |
| --- | --- | --- |
| GCK_exon_1_Foward | TCCACTTCAGAAGCCTACTG | Chr7:44228423-44228617 |
| GCK_exon_1_Reverse | TCAGATTCTGAGGCTCAAAC |  |
| HK1_E_Forward | c​a​g​g​c​t​g​g​t​a​c​t​c​g​a​g​a​c​a​c​ | Chr10:71108653-71108835 |
| HK1_E_Reverse | t​g​c​c​t​g​t​g​a​c​c​t​t​t​c​c​t​c​a​c​ |  |
| HK1_F_Forward | a​g​g​c​a​g​a​g​t​t​t​t​t​g​c​t​t​g​c​c | Chr10:71108620-71108804 |
| HK1_F_Reverse | c​c​a​c​t​g​a​a​g​c​t​g​g​g​a​g​a​a​c​c​ |  |

**c)**

|  | **Sequence (5’-3’)** |
| --- | --- |
| **Forward** | AGCCTGGGCAACAGAAAC |
| **Reverse** | GCTACAAGCTCAGCCTCTTTC |

**Supplementary Table 5:** Table describing all public genomic datasets used in this study.

| Accession | DOI | Description | Cell | Target |
| --- | --- | --- | --- | --- |
| GSE50386 | doi.org/10.1172/JCI66514 | ChIP-seq histone modifications in FACS sorted alpha, beta and exocrine cells | Alpha, Beta, Exocrine | H3K27me3, H3K4me3 |
| GSE149148 | doi.org/10.7554/eLife.59067 | ATAC-seq, ChIP-seq for TFs and Histone modifcations over pancreatic differentiation | ES, DE, GT, PP1, PP2 | CTCF, FOXA1, FOXA2, GATA4, GATA6, H3K27ac, H3K27me3, H3K4me1, H3K4me3, HNF6, PDX1, SOX9, ATAC |
| GSE118588 | doi.org/10.1016/j.celrep.2018.12.083 | ATAC-seq, ChIP-seq for TFs and Histone modifications in EndoC-BH1 cells | EndoC-BH1 | ATAC, CTCF, H3K27ac, H3K27me3, H3K36me3, H3K4me1, H3K4me3, H3K79me2,H3K9me2,H3K9me3 |
| E-MTAB-1919 | doi.org/10.1038/ng.2870 | ChIP-seq for TFs and Histone modifications in islets | Islets | CTCF, FOXA2, H2AZ, H3K27ac, H3K4me1, MAFB, NKX2_2, NKX6_1, PDX1 |
| E-MTAB-1990,  E-MTAB-3061 | doi.org/10.1038/ncb3160 | ChIP-seq for TFs and Histone modifications in pancreatic progenitors and pancreatic and liver buds | LiverBud, PP, PancreaticBud | FOXA2, GATA6, H3K27ac, H3K4me1, HNF1B, ONECUT1, PDX1, TEAD1 |
| GSE160472 | doi.org/10.1038/s41588-021-00823-0 | Single nuclei ATAC-seq in islets | Islets | ATAC |
| E087 | doi.org/10.1038/nature14248 | ChIP-seq for H3K27me3 in islets | Islets | H3K27me3 |
| GSE101207 | doi.org/10.1016/j.celrep.2019.02.043 | Single cell RNA-seq in islets | Islets |  |
| GSE167880 | doi.org/10.1038/s41587-022-01219-z | Single cell RNA-seq in sc-islets (data obtained from https://singlecell.broadinstitute.org/single_cell/study/SCP1526) | sc-Islets |  |
| GSE114297 | doi.org/10.2337/db18-0365 | Single cell RNA-seq in islets (data processed in above publication https://doi.org/10.1038/s41587-022-01219-z, and obtained https://singlecell.broadinstitute.org/single_cell/study/SCP1526) | Islets |  |
| GSE143783 | doi.org/10.1038/s42255-020-00314-2 | Single cell RNA-seq of beta-like cell differentiation, abundances projected on pseuodotime available in ref's Supplementary Table 4 | ES cell to beta-like cell differentiation |  |

**Supplementary Note**

**The International Congenital Hyperinsulinism Consortium**

Ivo Barić^1^, Liat de Vries^2,3^, Samar S. Hassan^4^, Khadija Nuzhat Humayun^5^, Floris Levy-Khademi^6^, Catarina Limbert^7^, Birgit Rami-Merhar^8^, Verónica Mericq^9^, Kristen Neville^10^, Yasmine Ouarezki^11^, Ana Tangari^12^, Charles Verge^10^, Esko Wiltshire^13^

1. Division for Genetics and Metabolic Diseases, Department of Pediatrics University Hospital Center Zagreb and University of Zagreb, School of Medicine, Kišpatićeva 12, 10000 Zagreb, Croatia
2. The Jesse Z and Sara Lea Shafer Institute for Endocrinology and Diabetes, National Center for Childhood Diabetes, Schneider Children’s Medical Center of Israel, Petach Tikva, Israel
3. Sackler Faculty of Medicine, Tel Aviv University, Tel Aviv, Israel
4. Department of Pediatric Endocrinology, Gaafar Ibn Auf Pediatric Tertiary Hospital, Khartoum, Sudan.
5. Department of Paediatrics and Child Health, Aga Khan University, Karachi, Pakistan
6. Division of Pediatric Endocrinology, Shaare Zedek Medical Center, Faculty of medicine, Hebrew University, Jerusalem, Israel
7. Unit for Paediatric Endocrinology and Diabetes, Centro Hospitalar e Universitário de Lisboa Central, Hospital Dona Estefania, Lisbon, Portugal
8. Medical University of Vienna, Department of Pediatric and Adolescent Medicine, Vienna, Austria
9. Department of Pediatrics, Institute of Maternal and Child Research, Clinica Las Condes, Santiago, Chile
10. Endocrinology, Sydney Children’s Hospital, Randwick and School of Women’s and Children’s Health, UNSW, Australia
11. EPH Hassan Badi, Algiers, Algeria
12. Saredo, Sanatorio Güemes, Pediatric Endocrinology Unit, Buenos Aires, Argentina
13. Department of Paediatrics and Child Health, University of Otago Wellington, Wellington, New Zealand
